# Cubital Tunnel Release Under Local and Regional Anesthesia: A Scoping Review

**DOI:** 10.1177/22925503251404418

**Published:** 2025-12-26

**Authors:** Madeline E. Hubbard, Amr AlMasri, Nasimul S. Huq

**Affiliations:** 1 Department of Medicine, 3710 McMaster University, Hamilton, ON, Canada; 2 Department of Surgery, 3710 McMaster University, Hamilton, ON, Canada; 3 Niagara Plastic Surgery Centre, Niagara Falls, ON, Canada

**Keywords:** cubital tunnel release, ulnar nerve decompression, local anesthesia, regional anesthesia, literature review, libération du tunnel cubital, décompression du nerf ulnaire, anesthésie locale, anesthésie régionale, revue de la littérature.

## Abstract

**Introduction:** Cubital tunnel syndrome (CuTS) occurs due to compression or traction of the ulnar nerve at the elbow. When conservative management fails, CuTS release (including decompression and transposition) can be performed. Recently, more research has investigated local anesthesia (LA) and regional anesthesia (RA) for CuTS release. The objective of this scoping review was to summarize current literature on the safety and efficacy of LA and RA for CuTS release. **Methods:** A scoping review was conducted following the PRISMA-ScR protocol and reporting guidelines. A search was conducted of MEDLINE, EMBASE, Web of Science and CINAHL based on the key concepts of CuTS release, and LA or RA. Covidence was used for abstract and full-text screening. **Results:** A total of 21 studies consisting of 1385 patients and 1406 elbows were included. Most studies were case series or cohort studies. Patients received LA in 15 studies (*n = *429 elbows), RA in nine studies (*n = *616 elbows) and general anesthesia (GA) in six studies (*n = *361 elbows). Complication rates after surgery were 2.9% for LA, 2.3% for RA, and 2.5% for GA. Overnight hospital stay was more often required in GA compared to RA. One study reported significantly less postoperative pain using LA compared to GA. Four studies reported preference for LA or had high satisfaction with their procedures. **Conclusions:** Regional and local anesthetic techniques are safe and feasible for CuTS release. They have similar complication rates to GA, but may offer additional benefits such as intraoperative feedback, and better postoperative pain management.

## Résumé

**Introduction :** Le syndrome du tunnel cubital (STC) survient en raison d’une compression ou d’une traction du nerf ulnaire au niveau du coude. En cas d’échec du traitement conservateur, la libération du tunnel cubital, comprenant décompression et transposition, peut être effectuée. Récemment, davantage de recherches ont étudié l’anesthésie locale (AL) et l’anesthésie régionale (AR) pour la libération du tunnel cubital. Cette revue exploratoire visait à synthétiser les données actuelles sur la sécurité et l’efficacité de l’AL et de l’AR dans la libération du tunnel cubital. **Méthodes :** Une revue exploratoire a été effectuée conformément au protocole PRISMA-ScR et à ses directives de rapport. Une recherche a été réalisée dans MEDLINE, EMBASE, Web of Science et CINAHL portant sur la libération du tunnel cubital, l’AL et l’AR. Le dépistage des résumés et textes complets a été réalisé à l’aide de Covidence. **Résultats :** Au total, 21 études, portant sur 1 385 patientes et 1 406 coudes, ont été incluses. La plupart des études étaient des séries de cas ou des études de cohorte. Les patientes ont reçu une AL dans 15 études (*n = *429 coudes), une AR dans 9 études (*n = *616 coudes) et une anesthésie générale (AG) dans 6 études (*n = *361 coudes). Les taux de complications post-opératoires étaient de 2,9% pour l’AL, 2,3% pour l’AR et 2,5% pour l’AG. Un séjour hospitalier d’une nuit était plus souvent nécessaire sous AG que sous AR. Une étude a montré une douleur postopératoire significativement réduite avec l’AL par rapport à l’AG. Quatre études ont signalé une préférence pour l’AL ou une forte satisfaction des patientes. **Conclusion :** Les techniques d’anesthésie régionale et locale sont sûres et réalisables pour la libération du tunnel cubital. Elles présentent des taux de complications similaires à l’AG, mais peuvent offrir des avantages supplémentaires tels que des retours peropératoires et une meilleure gestion de la douleur postopératoire.

## Introduction

Cubital tunnel syndrome (CuTS) is the second most common compressive neuropathy of the upper limb and it occurs when there is compression or traction of the ulnar nerve around the elbow.^1^ CuTS can be idiopathic or post-traumatic due to anatomic abnormalities or ulnar nerve subluxation.^2^ Management of CuTS can be nonsurgical, including nighttime elbow splinting, or surgical with ulnar nerve decompression, medial epicondylectomy, or transposition of the ulnar nerve.^1,2^

Surgical management of CuTS can be done under one of three different types of analgesia: general, regional, or local anesthesia (LA). General anesthesia has been classically used on patients who are not suitable for regional anesthesia (RA) and for patients who would otherwise be unable to psychologically tolerate being awake while in surgery.^3^ On the other hand, the latter two forms of anesthesia can be advantageous for the patient, surgeon, and the system overall.

Firstly, both RA and LA block nerve conduction in a designated area without providing systemic analgesia.^4,5^ Both of these techniques utilize a mixture of epinephrine, lidocaine, and other anesthetic compounds.^5,6^ For RA, and more specifically for upper extremity surgeries such as hand surgery, blockades are usually given as an axillary, supraclavicular, or intravenous blockade.^7^ Methods of LA include Wide-Awake Local Anesthesia with no Tourniquet (WALANT) and LA with a tourniquet, with or without sedation.

The use of LA and RA has been shown to be equally effective as the use of full body sedation with tourniquet for patients undergoing carpal tunnel surgery in metrics including patient satisfaction and comfort.^8,9^ In other upper limb surgeries, there have been no reported differences in complications between traditional anesthesia techniques and the WALANT technique.^6^ For patients undergoing cubital tunnel release, LA can be used in a minor procedures room and does not carry some of the risks associated with general anesthesia.^10^

Recently, more research has been devoted to investigating LA for cubital tunnel release. Currently, no comprehensive review exists that summarizes the safety and efficacy of local and RA for ulnar nerve decompression surgery. The primary objective of this scoping review is to summarize current literature on the safety and efficacy of local and RA for cubital tunnel release. This will be assessed based on postoperative complications, recovery time, pain control or other metrics of patient satisfaction for patients undergoing CuTS release with local or RA.

## Methods

A scoping review was conducted following PRISMA-ScR protocol and reporting guidelines.^11^ A review protocol was registered on Open Science Framework (OSF: https://osf.io/5c4vd).

### Search Strategy

After a preliminary search of the literature, a search strategy was developed based on two key concepts: cubital tunnel surgery and local or RA (Table S1). Inclusion criteria were studies that (1) reported on surgery for CuTS, (2) used local or RA for intraoperative anesthesia, (3) were published in a peer-reviewed journal, (4) included human subjects, and (5) were available in English. Studies were not limited by geographical range or date of publication. Databases included in this review were MEDLINE, EMBASE, Web of Science and CINAHL. The search was conducted on December 21, 2024 and EndNote was used to manage references.

### Study Screening

Two independent authors conducted title and abstract screening using Covidence. If a conflict arose, the authors discussed it with a third author. Full-text screening was conducted likewise.

### Data Extraction and Synthesis

Data was extracted to Google Sheets (Google LLC, Mountain View, CA, USA). Specific data included demographics, surgical details, anesthetic details, and post-operative outcomes (complications from surgery or anesthesia, recovery time, post-operative pain, etc.). Data were synthesized descriptively and presented in table format.

### Inter-Reviewer Agreement

Inter-reviewer agreement was assessed using the kappa (κ) statistic. *A priori* agreement was defined as: κ of 0.91–0.99 was almost perfect; κ of 0.71–0.90 was considerable; κ of 0.61–0.70 was high; κ of 0.41–0.60 was moderate; κ of 0.21–0.40 was fair; and κ of 0.20 or less was no agreement.^12^

### Quality Assessment

Quality assessment was based on the Methodological Index for Non-Randomized Studies (MINORS) criteria.^13^ Using the MINORS score and *a priori* classification, non-comparative studies could score a maximum of 16, and comparative studies could score a maximum of 24.^14^

## Results

### Study Characteristics

**Figure 1.**
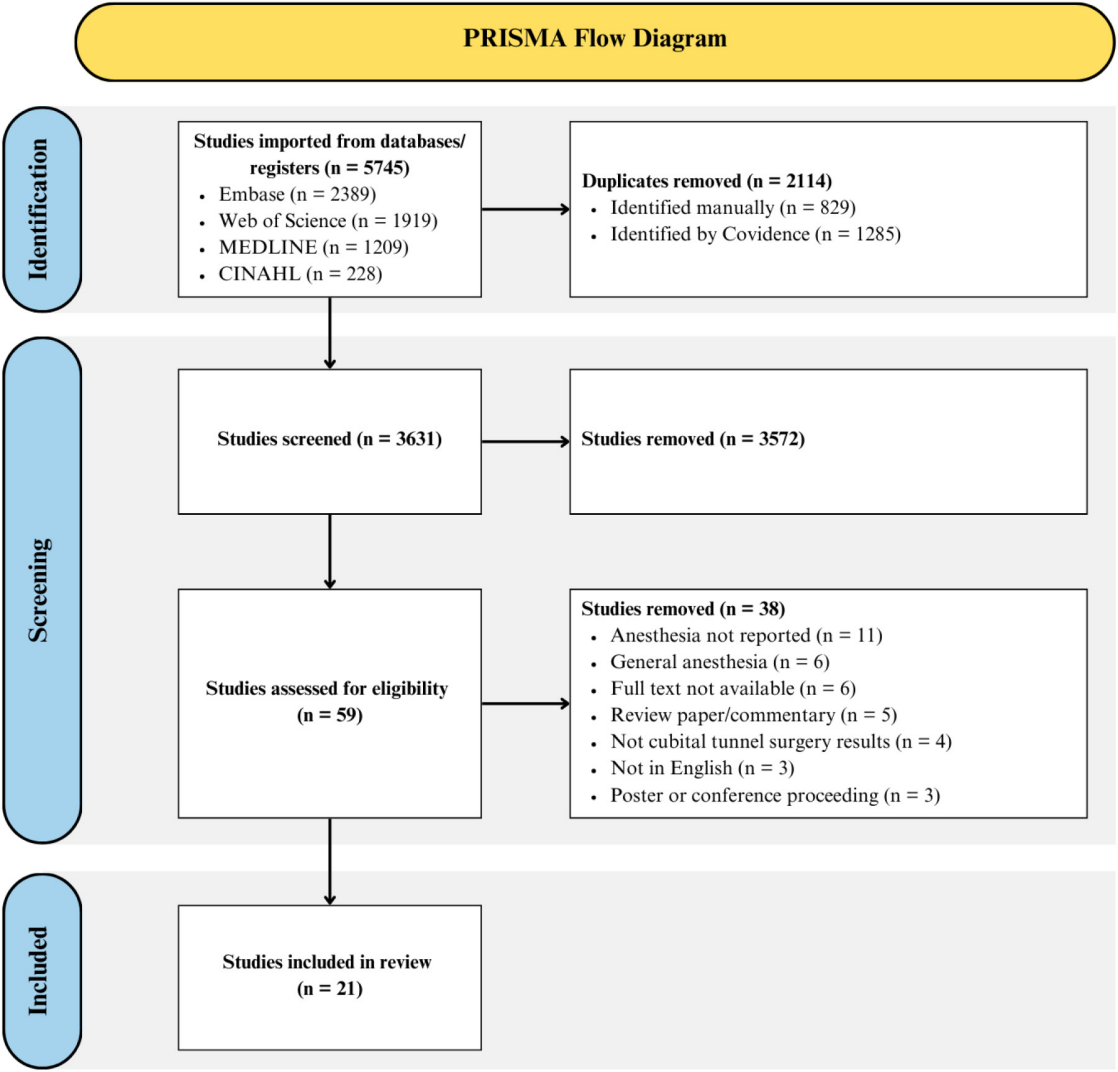
PRISMA Flow Diagram.

The inter-reviewer agreement was considerable (κ = 0.63) for title and abstract screening, and almost perfect (κ = 0.96) for full text screening. After screening, 21 studies were included in this review (Figure 1). Most of the studies were case series or cohort studies (Table 1). The mean MINORS score for comparative studies was 17.2/24 (range 14–21) with all studies being considered fair, good or high quality (Table 1). For non-comparative studies, the mean MINORS score was 10.2/16 (range = 7–12) with one study being considered low quality and the remaining being fair quality (Table 1).

**Table 1.** Demographic Data.

Author, year	Study design (level of evidence)	Anesthesia	Number of patients, *n*	Number of elbows, *n*	Mean (SD) [range] Age (years)	Female, *n* (%)	Mean (SD) [range] follow-up time (months)	MINORS score
Ajwani et al., 2019^15^	Cohort study (III)	LA + GA	23 - LA: 13, GA: 10	23 - LA: 13, GA: 10	63.5 - LA: 67, GA: 59	10 (43.2) - LA: 5 (38.0), GA: 5 (50.0)	LA: 40.1 (33.1) [2–93] wks, GA: 27.4 (36.7) [2–124] wks	17/24
Balevi and Ozturk, 2018^30^	Prospective case series (IV)	RA	15	15	43.6 (16.5) [15–72]	6 (40.0)	3.6 (6.2)	12/16
Bruggink et al., 2024^16^	Retrospective case series (IV)	LA	27	27	Median 50 [26–80]	13 (48.0)	1.25 [1.25–4]	10/16
Carlson Strother et al., 2023^31^	Retrospective cohort study (III)	RA + GA	80 - RA: NR, GA: NR	91 - RA: 55, GA: 36	55.5 - RA: 55.5 (15.4), GA: 55.5 (15.2)	38 (47.5) - RA: 22 (40.0), GA: 16 (44.4)	1.5	19/24
Debkowska et al., 2019^17^	Case Report (IV)	RA + LA	1	1	24	0, (0.0)	1.5	10/16
del Vecchio et al., 2014^18^	Retrospective cohort study (III)	LA + GA	50 - LA: 23, GA: 27	50 - LA: 23, GA: 27	47 [28–75]	26 (52.0) - LA: 16 (69.6), GA: 10 (37.0)	At least 12 mo.	18/24
Hebl et al., 2001^32^	Retrospective cohort study (III)	RA + GA	360 - RA: 100, GA: 260	360 - RA: 100, GA: 260	51.44 - RA: 55.7 (17.7), GA: 49.8 (17.4)	98 (27.3) - RA: 23 (23.0), GA: 75 (29.0)	NR	16/24
Jeon et al., 2010^19^	Retrospective case series (IV)	LA	62	66	49.7 [15–77]	36 (58.0)	23.9 [12–60]	11/16
Kang et al., 2019^20^	Retrospective cohort study (III)	LA + GA	42 - LA: 20, GA: 22	42 - LA: 20, GA: 22	53.34 - LA: 52.4 (12.8), GA: 54.2 (13.2)	11 (26.0) - LA: 5 (25.0), GA: 6 (27.0)	Up to 12 mo.	21/24
Lankester and Giddins, 2001^21^	Prospective case series (IV)	LA	20	20	59 [24–82]	10 (50.0)	16 [6–30]	12/16
Lavyne and Bell, 1982^22^	Retrospective case series (IV)	LA	19	20	54	4 (21.1)	[6–30]	11/16
LeRoux et al., 1990^23^	Retrospective case series (IV)	LA	46	51	59 [37–72]	0 (0.0)	17.8 [5–32]	10/16
Li et al., 2024^33^	Retrospective case series (IV)	RA	13	13	59 [45–73]	4 (30.8)	29 [14–66]	11/16
Mak et al., 2024^24^	Prospective case series (IV)	LA	29	29	57 [33–74]	8 (27.6)	At least 12 wks	12/16
Pagnotta et al., 2021^34^	Retrospective case series (IV)	RA	8	8	58.2 [33–74]	3 (37.5)	43.3 [24–71]	9/16
Roussel and Thirkannad, 2014^35^	Prospective cohort study (III)	RA	120	120	50.53	52 (43.3)	NR	14/24
Tarnasky et al., 2024^25^	Retrospective cohort study (III)	LA + RA + GA	244 - RA: 180, LA: 58, GA: 6	244 - RA: 180, LA: 58, GA: 6	median (IQR) - RA: 48.5 (38, 58), GA + LA: 52 (37, 60)	120 (49.2) - RA: 93 (51.7), GA + LA: 27 (42.2)	At least 3 mo.	18/24
Tchiloemba et al., 2023^26^	Case report (IV)	LA	1	1	19	1 (100)	1	8/16
Tosti and Rekant, 2022^27^	Surgical technique and case report (V)	LA	2	2	52	2 (100)	Case 1: 2 wks Case 2: NR	7/16
Vanaclocha et al., 2017^28^	Retrospective cohort study (III)	LA + RA	188 - LA: 64, RA: 124	188 - LA: 64, RA: 124	53.4 (12.1) [18–84]	73 (38.8) - LA: 23 (35.9), RA: 50 (40.3)	1511.1 (770.57) [310–4203] days	15/24
Yoshida et al., 2009^29^	Retrospective case series (IV)	LA	35	35	59.5 (9.9) [37–84]	NR	25.9 (11.2) [13–61]	10/16
Total	Case series *n = *10, Cohort study *n = *8, Other *n = *3	LA: 15, RA: 9, GA: 6	1385- LA: 419, RA: 561, GA: 325	1406- LA: 429, RA: 616, GA: 361	51.9 [15–84]- LA: 55.2, RA: 51.5, GA: 51.0	515 (38.2)- LA: 123 (37.7), RA: 253 (45.1), GA: 112 (35.2)	31.6 [0.5–138.2]- LA: 18.1, RA: 21.1, GA: 6.75	C: 17.2/24 [14–21], NC: 10.2/16 [7–12]

LA: local anesthesia; RA: regional anesthesia, GA: general anesthesia; NR: not reported; wks: weeks; mo: months; C: comparative studies; NC: non-comparative studies.

### Demographics

A total of 1385 patients were included in this review with 1406 surgeries (Table 1). Fifteen studies used LA for 419 patients (429 surgeries).^15–29^ RA was used in nine studies for 561 patients (616 surgeries).^17,25,28,30–35^ Six studies compared local or RA to general anesthesia for a total of 325 patients (361 surgeries) receiving general anesthesia.^15,18,20,25,31,32^ The mean age of included participants was 51.9 years, and 38.2% of participants were female (Table 1). The mean follow-up time was 31.6 months (Table 1).

### LA Only

Twelve studies assessed outcomes using LA alone for a total of 307 procedures. Among those studies, 10 used decompression surgery, while two performed a mixture of decompression and transposition (Table 2). Commonly used anesthetics were Lidocaine in eight (57%) studies, Bupivacaine in three (21%), Lignocaine in two (14%), and Marcaine in one (7%). In addition, epinephrine was used to supplement the anesthesia in nine (75%) of the 12 studies. Out of the 307 procedures completed with only LA, none were converted to general anesthesia and one case (0.3%) required intravenous sedation due to gross anxiety. Complications were reported in nine (2.9%) cases ranging from bleeding in three patients, two wound infections, two hematomas without further surgical intervention, one wound dehiscence, and one wound breakdown (Table 3). Compared to patients who received general anesthesia, those who received LA had less pain up to 48 h after surgery (*p* < .05) and less pain 2–12 h after surgery (*p* < .01).^20^

**Table 2.** Cubital Tunnel Surgery Under Local Anesthesia Only.

Study	Surgery *n* (%)	Anesthetic technique *n* (%)	Intra-op pain/anesthetics *n* (%)	Postoperative Pain Control	GA Conversion	Complications *n* (%)	Patient preference opinion	CuTS symptoms post-op *n* (%)
Ajwani et al., 2019^15^	Decompression	Bupivacaine + epinephrine: 13 (WALANT), GA: 10	NR	NR	None	NR	NR	Postoperative PRUNE scores similar (*p* = .97)
Bruggink et al., 2024^16^	Decompression	Lidocaine + epinephrine: 27 (WALANT)	NR	NR	NR	1 (3.7) superficial wound infection	NR	13 (48.1) had remaining symptoms, Pain: 7 (26), Paresthesia: 10 (37)
del Vecchio et al., 2014^18^	Decompression: • alone 11 (48) • + transposition 12 (52)	Lidocaine + epinephrine and bupivacaine: 23 (WALANT), GA: 27	Additional lidocaine w/ epinephrine was given if there was pain	Day 1: non-sig. Day 7: sig. worse pain GA compared to the LA (*p* < .05)	NR	1 (4.3) wound dehiscence LA 1 (4.3) wound dehiscence GA	LA group: sig. more likely to repeat the surgery compared to GA (*p* = .04). No sig. difference in satisfaction	NR
Jeon et al., 2010^19^	Decompression with minimal skin incision	Lidocaine: 66- + tourniquet, Diazepam and pethidine IV		NR	NR	2 (3.2) hematoma without further surgical intervention	NR	53 (80) symptom relief after surgery, 13 (20) remaining symptoms
Kang et al., 2019^20^	Decompression + Transposition (Only in subluxed patients)	Lidocaine + epinephrine: 20 (WALANT), GA: 22	Perioperative pain was 0.6 on VAS scale in LA group	LA: sig. less pain up to 48 h post op (*p* < .05). Post op tramadol injections requested by 3 (15) of LA, and 8 (36) of GA (*p* = .003)	None	None	NR	Both groups sig. improved in DASH, strength, and pinch test, No sig. difference between groups at 3 or 12 months after surgery.
Lankester and Giddins, 2001^21^	Decompression	Bupivacaine + lignocaine: 20- + tourniquet: 3 (15), + epinephrine in 17 (85)	Tourniquet pain limiting factor for 3 (15), so switched to epinephrine	NR	No GA, but 1 (5) required IV sedation for gross anxiety	None	16 (80) would have the operation under LA again. 4 (20) would not due to minor discomfort during the procedure or unease being awake.	18 (90): partial or complete improvement, 1 (5): gradual return of symptoms after 10 mo., 1 (5): no sig. improvement, no further deterioration.
Lavyne and Bell, 1982^22^	Decompression +/- epineurolysis	Lidocaine: 20- + Pre op Pentobarbital and atropine	NR	NR	NR	NR	NR	19 (95) symptomatic and objective relief, 1 (5) after 10 mo. required secondary epineurolysis
LeRoux et al., 1990^23^	Decompression	Lidocaine and Marcaine + epinephrine: 51- midazolam if necessary	NR	NR	NR	1 (2.2) infection 1 (2.2) hematoma w/ breakdown	NR	19 (37) full recovery, 22 (43) residual symptoms, 10 (20) no improvement
Mak et al., 2024^24^	Decompression w/ medial epicondylectomy	Lignocaine + epinephrine: 29- (WALANT)	Mean VAS /10- injection: 3.2, incision: 0.52, nerve dissection: 2.1, osteotomy: 0.9	Mean VAS /10- Immediate post op: 0.8	None	None	Mean satisfaction: 93.5/100	26 (89.7) subjective improvements in numbness
Tchiloemba et al., 2023^26^	Decompression	LA NR: 1 (WALANT)	NR	NR	None	None	NR	1 (100) pain improved, 1 (100) tremor, sensory, and motor symptoms persisted.
Tosti and Rekant, 2022^27^	Endoscopic decompression	Lidocaine + epinephrine: 2 (WALANT)- Tourniquet available	NR	NR	None	NR	NR	2 (100) symptoms resolved, 1 (50) pain minimal
Yoshida et al., 2009^29^	Endoscopic decompression	Lidocaine + epinephrine: 35	NR	NR	None	1 (3) converted to open (bleeding) 2 (6) returned to clinic in 8 h due to bleeding	NR	34 (97) improvement , 22 (63) no tingling, 32 (92) pain recovered, 31 (89) sensory disturbance recovered
Total *n = *307	Decompression: *n = *12, Transposition: *n = *2	WALANT *n = *132 (43), Tourniquet: *n = *69 (22), GA: *n = *59	Perioperative pain on VAS: range 0.5 - 3.2	Improved post-op pain in LA compared to GA: 2 studies	GA conversion: *n = *0 (0.0), Sedation: *n = *1 (0.3)	LA: 9 (2.9), GA: 1 (1.7)	Most patients would choose LA again over GA (2 studies), High satisfaction	Improvement: *n = *228 (74), Residual symptoms: *n = *62 (20), No improvement: *n = *16 (5.2)

LA: local anesthesia, GA: general anesthesia; NR: not reported, PRUNE: patient-rated ulnar nerve evaluation questionnaire; WALANT: Wide-Awake Local Anesthesia No Tourniquet; Op: operative.

**Table 3.** Cubital Tunnel Surgery Under Regional Anesthesia Only.

Study	Surgery	Anesthetic technique *n* (%)	Intra-op pain or anesthetics *n* (%)	GA conversion *n* (%)	Hospital stay *n* (%)	Complications *n* (%)	CuTS symptoms post-op *n* (%)
Balevi & Ozturk, 2018^30^	Modified decompression	AX: 15 (100)- + tourniquet	None	None	NR	None	Bishop scores: excellent 13 (86.7), good 1 (6.7), fair 1 (6.7)
Carlson Strother et al, 2023^31^	Decompression, +/–Guyon's release	SC: 30 (54.6), IC: 6 (10.9), AX: 19 (34.6), GA: 36- + tourniquet 97%	Pain: 6 (10.9), Intra-op LA: RA 5 (9.1), GA 11 (30.6)	6 (10.9)	1 (1.8) RA, 2 (5.6) GA *p* = .329	Total: RA 7 (12.7), GA 8 (22.2) *p* = 0.233; Pain: RA 5 (9.0), GA 4 (11.1); Edema: RA 4 (7.3), GA 2 (5.6); Rash: RA 1 (1.8); Hematoma: RA 1 (1.8); Other: GA 2 (5.6)	McGowan score: Not sig. different between groups at 6 wks . No change in score: RA 33 (60), GA 24 (66.7)
Hebl et al, 2001^32^	Transposition	AX: 100 (100), GA: 260, + tourniquet: RA 90%, GA 88%	NR	NR	RA: 49 (49), GA: 179 (69) *p* = 0.001	None	Paresthesia: RA 3 (3), GA 5 (2); Numbness: RA 2 (2), GA 5 (2); Worsening: RA 5 (5), GA 10 (4)
Li et al, 2024^33^	Decompression, transposition	BP: 13 (100)	NR	NR	NR	NR	Pain: mean [range] pre-op 3.7 [2–5], post-op 0 [0–0]
Pagnotta et al, 2021^34^	Decompression, adipofascial flap	BP: 8 (100) - Ultrasound-guided, + tourniquet	NR	NR	NR	NR	Pain: mean [range] pre-op: 6.1 [4–9], post-op 1.6 [0–3], *p* < .0001, McGowan score improved
Roussel & Thirkannad, 2014^35^	Decompression	SC: 40 (33.3), IC: 40 (33.3) AX: 40 (33.3) - Ultrasound-guided, + anxiolytics, + tourniquet	Failed blocks: AX 6 (20), IC 6 (20), SC 10 (33), RA touch-ups: 21 (23)	3 (3.3) GA or very deep sedation	NR	NR	NR
Total RA: *n = *311 GA: *n = *296	Decompression: *n = *5 Transposition: *n = *2	SC: 174 (55.9), IC: 46 (14.8), AX: 70 (22.5), BP: 21 (6.8), GA: 296	Pain: 6 (1.9), Additional anesthetic: RA 26 (8.4), GA 11 (3.7)	9 (2.9)	RA: 50 (16.1), GA: 181 (61.1)	RA: 7 (2.3), GA: 8 (2.7)	Improvement in elbow pain, No difference RA versus GA

RA: regional anesthesia, GA: general anesthesia; NR: not reported; SC: supraclavicular; IC: infraclavicular; AX: axillary, BP: brachial plexus.

After surgery with LA, cubital tunnel symptoms improved in 228 (74%) patients (Table 2). No improvement was reported in 16 (5.2%) patients. In 62 (20%) patients, there were residual symptoms such as pain, paresthesias, or numbness (Table 2). There were no significant differences in postoperative cubital tunnel symptoms in patients who received LA compared to general anesthesia.^15,20^ Patients were more likely to have improvements if their preoperative symptoms were mild, had an onset < 1 year prior to surgery, and did not involve muscle atrophy.^23^ Patient preference was reported in favour of LA in two studies, and another reported high patient satisfaction.^18,21,24^ Notably, 20% of patients in one study would not repeat the procedure with LA due to minor discomfort or unease related to being awake in the operating room.^21^

### RA Only

In six studies that assessed RA for cubital tunnel surgery, supraclavicular blocks were the most common, being performed in 55.9% of patients (Table 3). Out of 311 patients who received RA, there were 6 (1.9%) reports of intra-operative pain. Additional anesthetics, including intraoperative LA and RA touch ups, were required in 26 (8.4%) of patients (Table 4). In comparison, 11 (3.7%) control patients receiving general anesthesia were given additional intraoperative local anesthesia. Overall, nine (2.9%) of patients who received RA were converted to general anesthesia or required very deep sedation, largely due to pain.

**Table 4.** Cubital Tunnel Surgery Under Regional Anesthesia and Local Anesthesia.

Study	Surgery	Anesthetic *n* (%)	Intra-op pain or GA conversion *n* (%)	Hospital stay *n* (%)	Patient preference	Complications	CuTS symptoms post-op
Debkowska et al, 2019^17^	Decompression	SC: 1 (100), LA: lidocaine + epinephrine	NR	NR	NR	NR	Strength improved from 4/5 pre-op to 5/5 post-op
Tarnasky et al, 2024^25^	Decompression, transposition and/or neurolysis	RA: 180 - SC: 117 (83.6), IC: 7 (5.0), AX: 16 (11.4); LA + GA: 64 - LA: 58 (90.6), GA: 6 (9.4)	NR	NR	NR	New median nerve deficit: RA 1 (0.6%), GA + LA 2 (3.1%), *p* = .17	>70% improved, not different between groups (*p* = .14). Persistent neurologic symptoms: RA: 20.6%, GA + LA 25%, *p* = .48. Persistent neuropathic pain: RA 5.6%, GA + LA 18.8%, *p* = .004.
Vanaclocha et al, 2017^28^	Decompression (lateral decubitus vs supine) or transposition (supine, subcutaneous, submuscular)	LA: lateral decubitus group 64, RA: all others 124	NR	Outpatient for lateral with LA	LA comfortable for patients and surgeon	Larger incision in RA (supine) groups associated with medial antebrachial cutaneous nerve deficits. Smaller incision in LA (lateral decubitus): no nerve deficit	Faster return to work in lateral decubitus with LA. Better post-op Gabel, Amadio, Bishop, PRUNE, DASH, and Michigan scores in decompression lateral decubitus LA compared to supine RA.

LA: local anesthesia; RA: regional anesthesia, GA: general anesthesia; NR: not reported; SC: supraclavicular; IC: infraclavicular; AX: axillary; BP: brachial plexus.

One study reported no difference in the number of patients requiring an overnight hospital stay for post-operative pain control between regional and general anesthesia.^31^ However, another study reported a significantly lower rate of overnight hospital stay in patients who received RA compared to general anesthesia (Table 3).^32^ Minimal complications were reported post-operatively for patients receiving cubital tunnel surgery under RA (Table 3). Complications were reported at a similar rate (2.3–2.7%) between patients receiving regional and general anesthesia, including pain, edema, rash and hematoma (Table 3). Improvements in CuTS symptoms, including elbow pain, were similar between patients receiving regional and general anesthesia (Table 3).

### Local and RA

In a case report of a hemophiliac patient undergoing ulnar nerve decompression at the elbow, the patient received a supraclavicular nerve block plus local infiltration of lidocaine and epinephrine.^17^ There were no complications related to the anesthesia or surgery, and the patient's cubital tunnel symptoms improved post-operatively (Table 4).

Another study compared patients who received a peripheral nerve block versus patients who received either local or general anesthesia for decompression, transposition and/or neurolysis of the ulnar nerve at the elbow.^25^ There was less persistent neuropathic pain in patients who received RA compared to patients who received either general or local anesthesia (Table 4). Otherwise, the rate of improvement was similar between RA and general or LA.^25^

A third study compared ulnar nerve decompression and transposition in the lateral decubitus and supine positions.^28^ Patients who had ulnar nerve decompression in the lateral decubitus position received LA, whereas all other patients received RA (Table 4). Patients in the lateral decubitus with LA group had smaller incisions, fewer postoperative nerve deficits and a faster return to work compared to patients who had surgery in a supine position with RA.^28^ Patients and surgeons reported being comfortable using LA during the surgery.

## Discussion

Overall, the current scoping review shows that regional and local anesthetic techniques are safe and feasible for cubital tunnel release. Complications for cubital tunnel release were reported in 2.9% of patients receiving LA, 2.3% of patients receiving RA and 2.5% of control participants with general anesthesia. These rates of complications are comparable to previous research reporting a complication rate of 3% across eight different surgical techniques for cubital tunnel decompression.^36^ The main complications reported were pain, edema, hematoma, wound dehiscence and infection.

Several benefits of LA have been suggested by previous research.^10^ In the current review, 43% of procedures with LA were conducted with WALANT. This surgical technique allows for patient-surgeon interaction during surgery, which can provide real-time feedback as to the effectiveness of the procedure.^37^ In addition, local anesthetic techniques such as WALANT do not necessarily require preoperative fasting or the cessation of home medications, which allows patients with contraindications for general anesthesia to receive surgery.^38^ On an economical side, LA is less resource intensive than general anesthesia as it can be done in an office-based or minor procedures setting rather than in an operating room.^10,38^ As well, patients recuperate faster after surgery, which reduces the need for postoperative recovery rooms.^38^ This allows surgeons to use their outpatient offices for surgeries, helping reduce not only the load on the healthcare system but also the cost associated with the surgery.^8^ Previous studies have shown that WALANT is equally as effective as other traditional anesthesia techniques for carpal tunnel release and other upper limb surgeries.^6,39^

In the current study, most patients were satisfied with LA and would choose it again over general anesthesia. As well, patients who had LA had lower postoperative pain compared to those who had general anesthesia. Patients who would not have the procedure again complained of minor discomfort during the procedure or unease at being awake in the operating room. Patients most commonly have discomfort during LA because of either incomplete tumescence of the operative field due to insufficient volume of LA, or not waiting a sufficient length of time for the LA to take effect prior to commencing.^40^ Anxiety during the procedure could be mediated by administering additional anxiolytics as needed or modifying the operating room environment, such as by having relaxing music. As well, patient selection for local and RA should consider the patient's baseline anxiety, to ensure that patients are suitable candidates.

When compared to general anesthesia, RA has been shown to reduce postoperative nausea, vomiting and chronic pain.^41^ Some evidence suggests that RA also reduces postoperative infection rates.^42^ RA can lead to decreased blood loss during surgery, and helps reduce the metabolic stress response to surgery.^43^ More specifically, RA reduce the likelihood of mortality due to deep vein thrombosis, pulmonary embolisms, transfusion requirements, pneumonia, or respiratory depression.^44^ The findings of the current review build on previous research by showing that RA also decreases the likelihood of patients requiring an overnight hospital stay.

On the other hand, both of these methods have associated disadvantages. One of the most severe potential complications with the use of local or regional anesthetics is Local Anesthetic Systemic Toxicity (LAST).^45^ LAST is a potentially lethal complication that involves a collection of progressively worsening neurological or cardiovascular symptoms due to anesthetic medication entering circulation.^46^ The risk of LAST in tumescent LA is negligible if long-lasting bupivacaine or ropivacaine are avoided and the maximum recommended dosage limit of 7 mg/kg of lidocaine with epinephrine is followed.^47^ The risk of LAST in regional nerve blocks can be reduced by almost four-fold with the use of an ultrasound.^48^

Another possible complication of WALANT is vasovagal syncopal episodes during local injection.^49^ In the current review, no such events were reported. In patients who had LA with a tourniquet, pain related to the tourniquet was a limiting factor. This can be avoided by using epinephrine to control surgical bleeding rather than a tourniquet. In patients who received RA, 2.9% were converted to general anesthesia or required deep sedation. Patients should be thoughtfully selected for local and RA to ensure they are suitable candidates, which can reduce the risk of requiring general anesthesia conversion.

Among the key strengths of this scoping review is the broad inclusion criteria that allowed for the inclusion of various types of literature and study designs, such as case series, cohort studies, and case reports. Another strength of this scoping review is that it assesses the methodological rigor and risk of bias within the included studies. This scoping review can provide guidance to surgeons and anesthetists planning for cubital tunnel surgery. By offering local or RA to appropriate patients, this can lead to changes in practice that improve patient postoperative pain and decrease hospital admissions.

A limitation of this scoping review is the limited amount of studies available to analyze. Most studies were case series or cohort studies and no randomized controlled trials were available. In addition, the results of included studies were predominantly descriptive, precluding the ability to synthesize data statistically. Future research should involve high-quality randomized controlled trials comparing cubital tunnel release under local, regional and general anesthesia to confirm the findings of this study.

## Conclusion

In conclusion, cubital tunnel release under local and RA is safe and feasible. These anesthetic techniques have similar complication and success rates as general anesthesia. They offer the benefits of intraoperative feedback, improved postoperative pain control, and lower rates of overnight hospital stay. Future research should consider randomized controlled trials to further evaluate these findings.

## Supplemental Material

sj-docx-1-psg-10.1177_22925503251404418 - Supplemental material for Cubital Tunnel Release Under Local and Regional Anesthesia: A Scoping Review Supplemental material, sj-docx-1-psg-10.1177_22925503251404418 for Cubital Tunnel Release Under Local and Regional Anesthesia: A Scoping Review by Madeline E. Hubbard, Amr AlMasri and Nasimul S. Huq in Plastic Surgery

sj-docx-2-psg-10.1177_22925503251404418 - Supplemental material for Cubital Tunnel Release Under Local and Regional Anesthesia: A Scoping Review Supplemental material, sj-docx-2-psg-10.1177_22925503251404418 for Cubital Tunnel Release Under Local and Regional Anesthesia: A Scoping Review by Madeline E. Hubbard, Amr AlMasri and Nasimul S. Huq in Plastic Surgery
