# Supplementary material for: Cubital Tunnel Release Under Local and Regional Anesthesia: A Scoping Review
Source: Plast Surg (Oakv). 2025 Dec 26:22925503251404418. Online ahead of print. doi: 10.1177/22925503251404418 (PMC12743009; doi:10.1177/22925503251404418)
Supplement: sj-docx-1-psg-10.1177_22925503251404418 - Supplemental material for Cubital Tunnel Release Under Local and Regional Anesthesia: A Scoping Review [file sj-docx-1-psg-10.1177_22925503251404418.docx]

**Table S1.** Search strategy sample of Web of Science

| **Concept** | **Search Terms** |
| --- | --- |
| Cubital tunnel release | 1. “Cubital tunnel” or “cubital tunnel syndrome” or cubital* or “ulnar nerve entrapment” or “ulnar nerve” or ulnar* or “sulcus ulnaris” or “sulcus ulnaris syndrome” or “retrocondylar groove” or “retrocondylar groove syndrome” 2. Release or decompression or transposition or surgery |
| Local anesthesia | 1. Anesthe* or local or nerve block or lidocaine or bupivacaine or ropivacaine or levobupivacine 2. WALANT or “wide awake local anesthesia no tourniquet” |
|  | 1. 1 AND 2 (n =18,896 ) 2. 3 OR 4 (n = 6,312,619) |
|  | 1. **5 AND 6 (n = 1,919)** |
